# The Enigmatic Conservation of a Rap1 Binding Site in the *Saccharomyces cerevisiae HMR-E* Silencer

**DOI:** 10.1534/g3.112.004077

**Published:** 2012-12-01

**Authors:** Leonid Teytelman, Erin A. Osborne Nishimura, Bilge Özaydin, Michael B. Eisen, Jasper Rine

**Affiliations:** * Department of Molecular and Cell Biology and California Institute for Quantitative Biosciences; † Howard Hughes Medical Institute, University of California, Berkeley, California 94720

**Keywords:** silencing, *sensu stricto*, Rap1, genomics, transcription factors

## Abstract

Silencing at the *HMR* and *HML* loci in *Saccharomyces cerevisiae* requires recruitment of Sir proteins to the *HML* and *HMR* silencers. The silencers are regulatory sites flanking both loci and consisting of binding sites for the Rap1, Abf1, and ORC proteins, each of which also functions at hundreds of sites throughout the genome in processes unrelated to silencing. Interestingly, the sequence of the binding site for Rap1 at the silencers is distinct from the genome-wide binding profile of Rap1, being a weaker match to the consensus, and indeed is bound with low affinity relative to the consensus sequence. Remarkably, this low-affinity Rap1 binding site variant was conserved among silencers of the *sensu stricto Saccharomyces* species, maintained as a poor match to the Rap1 genome-wide consensus sequence in all of them. We tested multiple predictions about the possible role of this binding-site variant in silencing by substituting the native Rap1 binding site at the *HMR-E* silencer with the genome-wide consensus sequence for Rap1. Contrary to the predictions from the current models of Rap1, we found no influence of the Rap1 binding site version on the kinetics of establishing silencing, nor on the maintenance of silencing, nor the extent of silencing. We further explored implications of these findings with regard to prevention of ectopic silencing, and deduced that the selective pressure for the unprecedented conservation of this binding site variant may not be related to silencing.

Similar to metazoan heterochromatin, *Saccharomyces cerevisiae’s* silenced chromatin occupies regions of the genome in which gene expression is inhibited. Silencing in yeast is not essential for viability but could lead to lethality were it misregulated (Ehrentraut *et al.* 2010). Several studies address how silenced chromatin domains are restricted from spreading into neighboring regions and shutting off useful or essential genes (Donze *et al.* 1999; Ehrentraut *et al.* 2010; Meneghini *et al.* 2003). However, little is known in *Saccharomyces cerevisiae* about mechanisms that prevent silencing at inappropriate regions, or conversely, how silencers enforce the specific locations of heterochromatin. This problem is particularly relevant in *S. cerevisiae* because binding sites for ORC, Rap1, and Abf1, which collectively make up the silencers, are individually common throughout the genome, mediating their individual specialized functions.

In *S. cerevisiae*, the best-studied examples of silenced chromatin occur at the cryptic mating loci *HML* and *HMR*. The silencing complex, consisting of the Sir1, Sir2, Sir3, and Sir4 proteins, is targeted to silencers—regulatory sites flanking both *HML* and *HMR*. The targeting occurs via interactions between and among Sir proteins and the ORC, Rap1, and Abf1 proteins bound to silencers. Once bound to the silencer, the Sir proteins initiate the formation of a specialized chromatin structure that prevents transcription [reviewed in (Rusche *et al.* 2003)]. A synthetic silencer consisting only of binding sites for Orc1, Abf1, and Rap1 is fully capable of silencing *HMR*, indicating that no additional DNA elements are needed to silence *HMR-E* (McNally and Rine 1991). However, the natural *HMR-E* silencer is stronger than the synthetic one in that silencing of *HMR* is maintained by the natural *HMR-E* silencer even after mutating of any one of the three binding sites (Brand *et al.* 1987). Synthetic silencers are more sensitive to the loss of silencing function upon mutation of individual silencer elements (McNally and Rine 1991; Weber and Ehrenhofer-Murray 2010). Furthermore, arrays of Rap1 binding sites are able to establish Sir-based silencing at telomeres, and synthetic arrays of Rap1 binding sites exhibit weak silencing ability (Cockell *et al.* 1995; Hecht *et al.* 1996; Stavenhagen and Zakian 1998).

The proteins that directly bind silencer DNA sequences all have additional individual roles in euchromatin. The Abf1 and Rap1 proteins are transcription factors involved in regulating the transcription of hundreds of genes (Lee *et al.* 2002; Lieb *et al.* 2001). The Origin Recognition Complex (ORC) binds to each of the approximately 400 yeast origins of replication and plays an essential role in initiating DNA replication (Breier *et al.* 2004; Poloumienko *et al.* 2001; Wyrick *et al.* 2001). Genome-wide binding profiles of the Sir2, Sir3, and Sir4 proteins and expression profiles of *sir−* mutants indicate that they typically are present only at *HML*, *HMR*, and chromosome ends, where silencing also takes place (Barton and Kaback 2006; Gottschling *et al.* 1990; Lieb *et al.* 2001; Vega-Palas *et al.* 2000; Wyrick *et al.* 1999). Silencing appears to be tightly restricted to the aforementioned regions, and reports of Sir-mediated silencing of euchromatic genes have proven unreliable (Marchfelder *et al.* 2003).

The lack of concordance in the genomic distributions of ORC, Rap1, and Abf1 and of the Sir complex raises two questions that are fundamental to the organization of euchromatin and heterochromatin: (1) How does the cell prevent ectopic silencing from happening throughout the genome, for example, wherever Rap1 and Abf1 bind? (2) What is special about the binding sites for these proteins or their organization in the silencers that results in the recruitment of Sir proteins instead of transcription proteins or DNA replication components? We addressed these questions by perturbation of the *HMR*-*E* silencer in *S. cerevisiae*, by studying the evolution of silencers across the closely related *sensu stricto* species, and by analyzing the genomic distributions of the individual binding sites of Rap1 and Abf1 in budding yeasts. In particular we asked whether the ultra-conserved Rap1 binding site in silencers, if substituted with the genome-wide consensus binding site of Rap1, could maintain its native level of performance in silencing.

## Materials and Methods

### Strains and primers

Yeast strains and primers used in this study are listed in Table 1 and Table 2.

**Table 1:** Yeast strains

Strain	Genotype	Source
JRY2334	*MATa ade2-1 can1-100 his3-11 leu2-3,112 trp1-1 ura3-1*	R. Rothstein
JRY3009	*MATα ade2-1 can1-100 his3-11 leu2-3,112 trp1-1 ura3-1*	R. Rothstein
JRY4565	*MATα ade2-1 can1-100 his3-11 leu2-3,112 trp1-1 ura3 -1 sir2*Δ::*TRP1*	
JRY8991	*MATα ade2-1 can1-100 his3-11 leu2-3,112 trp1-1 ura3-1 HMR*-eΔ::*KL_URA3*	
JRY8992	*MATα ade2-1 can1-100 his3-11 leu2-3,112 trp1-1 ura3-1 HMR*-eΔ::*KL_URA3*	
JRY8994	*MATα ade2-1 can1-100 his3-11 leu2-3,112 trp1-1 ura3 -1 consensus-Rap1-bs-HMR-E*	
JRY8995	*MATα ade2-1 can1-100 his3-11 leu2-3,112 trp1-1 ura3 -1 consensus-Rap1-bs-HMR-E*	
JRY9017	*MATα ade2-1 his3-11 trp1-1 leu2-3,112 ura3-1 can1-100 sir1 (13 -641*Δ*)*	
JRY9018	*MATα ade2-1 his3-11 trp1-1 leu2-3,112 ura3-1 can1-100 sir1 (13 -641*Δ*)*	
JRY9019	*MATα ade2-1 his3-11 trp1-1 leu2-3,112 ura3-1 can1-100 sir1 (13 -641*Δ*) consensus-Rap1-bs-HMR-E*	
JRY9020	*MATα ade2-1 his3-11 trp1-1 leu2-3,112 ura3-1 can1-100 sir1 (13 -641*Δ*) consensus-Rap1-bs-HMR-E*	
JRY9021	*MATα ade2-1 his3-11 trp1-1 leu2-3,112 ura3-1 can1-100 RAP1-MYC*::*HYG*	
JRY9022	*MATα ade2-1 his3-11 trp1-1 leu2-3,112 ura3-1 can1-100 RAP1-MYC*::*HYG*	
JRY9023	*MATα ade2-1 his3-11 trp1-1 leu2-3,112 ura3-1 can1-100 RAP1-MYC*::*HYG consensus-Rap1-bs-HMR-E*	
JRY9024	*MATα ade2-1 his3-11 trp1-1 leu2-3,112 ura3-1 can1-100 RAP1-MYC*::*HYG consensus-Rap1-bs-HMR-E*	

**Table 2:** Oligonucleotide primers

Oligo Name	Sequence
oBO29 (a1 -F)	tggatgatatttgtagtatggcgga
oBO30 (a1 -R)	tccctttgggctcttctctt
act1-F	tgtccttgtactcttccggt
act1-R	ccggccaaatcgattctcaa
Sc HMR-E 3f	cgaacgatccccgtccaagttatg
Sc HMR-E 2r	tcggaatcgagaatcttcgtaatgc
Sc SEN1 f1	accaaaggtggtaatgttgatgtc
ScSEN1r1	gggaggcgatggtttagcctgtag
Sc TEL VI R f1	ggatatgtcaaaattggatacgcttatg
Sc TEL VI R r1	ctatagttgattatagatcctcaatgatc
Sc HMR-E flanking left (for sequencing)	tccttcacatcatgaaatataa
Sc HMR-E flanking right (for sequencing)	accaggagtacctgcgcttattct

### Strain construction

Site-directed mutagenesis (Goldstein and McCusker 1999; Longtine *et al.* 1998) was used to replace the AAACCCATCAACC *HMR-E* native Rap1 binding site with the genome-wide Rap1 consensus sequence: ACACCCATACATT. DNA sequencing confirmed the changes. To introduce this altered *HMR*-*E* sequence into the genome, the native *HMR*-*E* was first replaced with the *Kluyveromyces lactis URA3* (pUG72) in JRY3009 (Gueldener *et al.* 2002), resulting in isogenic *HMR*-eΔ::*KL_URA3* strains (JRY8991, JRY8992). The *HMR*-*E* sequence containing the Rap1 consensus sequence was then transformed into this strain and successful replacements were identified by counter selection against *URA3* using 5-Fluoro-orotic acid, producing the *consensus-Rap1-HMR-E* strains (JRY8994, JRY8995). Correct integration was confirmed by PCR and sequencing with primers flanking *HMR-E*.

The *sir1* mutant allele was generated by replacing all but 12 codons of the *SIR1* ORF with the *Kluyveromyces lactis URA3* [pUG72 (Gueldener *et al.* 2002)]. The resulting *sir1* mutants phenocopied cells with the *sir1* null. Rap1 was tagged on the C-terminus with 13xMyc::KanMX (Longtine *et al.* 1998) and transformed into JRY2334. This strain was crossed into JRY8994 to create JRY9021 and JRY9023. Because Rap1 is essential, the viability of cells with only the tagged form of Rap1 established that the tagged Rap1 was functional.

### Assay for the establishment of silencing

The parental strain (JRY3009) and the two independent mutant strains bearing the same *consensus*-Rap1-*HMR*-*E* mutations (JRY8994, JRY8995) were grown overnight in the presence of 10mM nicotinamide, a potent inhibitor of the Sir2 deacetylase, in 100 mL of rich medium at 30° to a density of approximately 2 × 10^7^ cells per milliliter. Each of the three cultures was harvested by centrifugation, and the media with nicotinamide removed and replaced with 100 mL of the rich media. Immediately after resuspending the cells, 10-mL samples of each culture were pelleted, frozen in liquid nitrogen, and then stored at −80°. This sample represented time point 0, postnicotinamide. Subsequently, after 7, 17, 24, 32, and 45 min of incubation at 30°, aliquots of cells were collected in the same manner. The 10-mL samples were extracted without dilution of the main cultures. An additional sample of silenced cells (JRY3009), grown overnight without nicotinamide, served as reference of fully-silenced ***a****1* levels. After collection, all samples are processed with the QIAGEN RNeasy Kit to extract the RNA (mechanical disruption protocol with on-column DNase digestion). Oligo-dT primer-directed cDNA was synthesized using the Super-Script III First-Strand Synthesis System for RT-PCR kit from Invitrogen. Quantitative polymerase chain reaction (qPCR) analysis was done in triplicate on each RNA preparation. qPCR was performed on a MX3000P machine (Stratagene) using SYBR GreenER qPCR super mix (Invitrogen). The cDNA levels were then measured for the ***a****1* gene at *HMR* and normalized to *ACT1* control cDNA measurements.

### Abf1 and Rap1 binding site conservation

Potential proto-silencers of *Saccharomyces*, defined as intergenic regions in which the Rap1 and Abf1 binding sites occur within 50 base pairs of each other, were identified using the map of Rap1 and Abf1 binding site matches from published work (MacIsaac *et al.* 2006). Percent of all binding-site matches conserved in three or more *sensu stricto* species was calculated for the binding sites genome-wide and in the proto-silencers, again using the conservation data from the aforementioned study.

### Abf1 and Rap1 binding site frequency by transcription-factor-specific intergenic regions

Transcription-factor-specific intergenic regions were defined based on the performed chromatin immunoprecipitation (ChIP)-chip dataset (Harbison *et al.* 2004). Only factors with *P* < 0.05 binding to 60 or more distinct intergenic regions were considered. For each transcription factor, we identified Abf1 binding site matches by using PATSER (Hertz and Stormo 1999), by searching with the Abf1 position weight matrix (Harbison *et al.* 2004) in all of the intergenic regions bound by the given factor. Matches with PATSER p-value < 10^−9^ were selected. Abf1-site frequency per transcription factor was calculated as the total number of PATSER matches, divided by the sum of the lengths of the intergenic regions bound by the transcription factor, and multiplied by 10,000 (resulting in number of Abf1-matches per 10 kilobases of sequence). The same approach was used for Rap1 binding-site frequencies.

### Binding-site profiles for Rap1 and Abf1 in *sensu stricto* species

For the *sensu stricto* species analysis, orthologous intergenic regions were identified by best-reciprocal-BLAST hits of the two flanking genes between *S. cerevisiae* and each of the other four species. For each species individually, motif searches were performed with MEME (Bailey and Elkan 1995) on orthologous regions corresponding to *S. cerevisiae* Rap1- or Abf1-bound intergenic regions, based on the ChIP-chip dataset (Harbison *et al.* 2004). Graphical position-weight matrices were constructed from the MEME matches with WebLogo (Crooks *et al.* 2004).

## Results

### Ultraconservation of the *HMR*-E Rap1 binding-site variant in *sensu stricto* species

The Rap1 binding site in the *HMR*-*E* silencer is a poor match to the typical sequence that Rap1 binds, and the *in vitro* affinity of Rap1 for the silencer version of the Rap1 binding sequence is approximately ten-fold lower than its affinity for the consensus sequence (Taylor *et al.* 2000). Because the binding sites at *HMR*-*E* are partially overlapping for silencing function (Brand *et al.* 1987; Kimmerly and Rine 1987), the presence of the weak Rap1 binding site *per se* was not striking. However, this variant became puzzling and conspicuous in the context of the level of divergence of the sequences flanking *HML* and *HMR* across *S. cerevisiae*’s closely related *sensu stricto* species (*S. paradoxus*, *S. mikatae*, *S. kudriavzevii*, *S. bayanus*). Compared with most of the genome, these sequences evolved much faster within and between species. We identified the conserved *HMR-E* in these species and found that deletion of the putative silencer from *S. bayanus*, the most distant of these species from *S. cerevisiae*, led to loss of silencing, confirming that these sequences had a conserved role in silencing (Teytelman *et al.* 2008).

**Figure 1:**
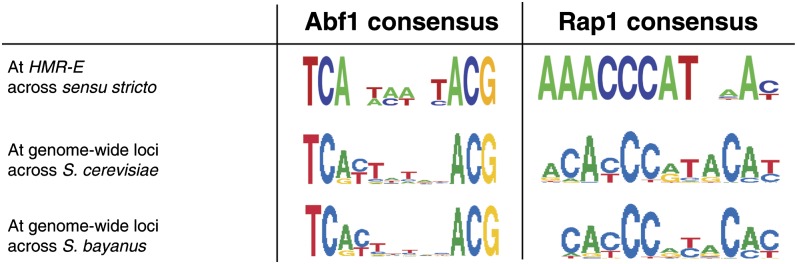
Conservation of *HMR-E* Rap1 and Abf1 binding sites in *sensu stricto* species. The Abf1 and Rap1 consensus sequences are depicted. Abf1 and Rap1 binding sites at silencers as they occur across all *sensu stricto* species are shown as compared to the genome-wide consensus sequences from both S. *cerevisiae* and *S*. *bayanus*.

We then compared the conservation of the *HMR*-*E* binding sites for Rap1 and Abf1 to their genome-wide profiles from *S. cerevisiae* ChIP-chip studies (Harbison *et al.* 2004). The Abf1 binding site within silencers closely matched the general profile for Abf1. In contrast, the Rap1 binding site at *HMR-E* (AAAACCCATCAAC) was virtually invariant among these species, conserved as a poor match to the inferred genome-wide Rap1 binding profiles in each of the *sensu stricto* species (Figure 1). This level of conservation of the Rap1 binding site was striking in light of the accelerated base-pair substitutions around and between the Rap1 and Abf1 binding sites (Teytelman *et al.* 2008). In addition, the Rap1 binding site at *HML-E* (AAAACCCATTCAT) is similar to the *HMR-E* binding site and is also a weak match to the Rap1 consensus sequence. The apparent constraint on the Rap1 binding site variant at *HMR-E* strongly suggested that this specific Rap1 binding-site sequence offered some quality to the silencer that closer matches to the consensus sequence could not.

### Rap1 consensus binding site at *HMR-E* was fully functional in silencing

Piña and colleagues have suggested that the particular site bound by Rap1 may induce the protein into a confirmation that is biased either to act as an activator or as a recruiter of Sir proteins (Pina *et al.* 2003). The latter scenario would be similar to the glucocorticoid receptor binding sites in human cells which act as ligands to induce site-specific functions of the receptor. The DNA variants of the glucocorticoid receptor binding site impact the confirmation and regulatory activity of the receptor, and replacing a weak site with the higher-affinity consensus alters the transcriptional response to the hormone (Meijsing *et al.* 2009).

**Figure 2:**
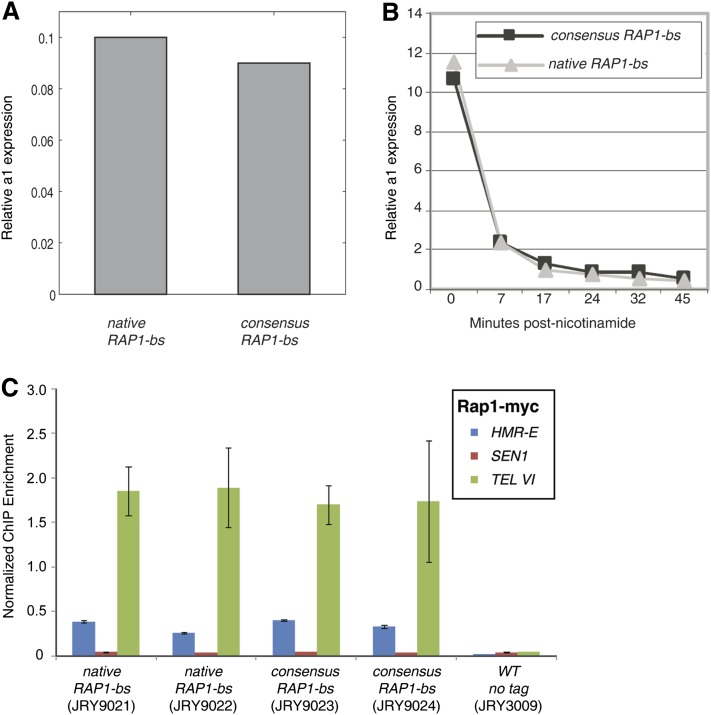
Robust silencing by the consensus Rap1 binding site at *HMR-E*. (A) Steady-state RT-qPCR measurements of *HMR*-**a1** transcript levels, normalized to *ACT1* control. (B) *HMR*-**a1** levels after removal of the nicotinamide silencing block. The gray curve illustrates the levels for the native *HMR-E* parental strain (JRY3009) and the black curve for the genomic consensus version of the Rap1 binding site (bs) at *HMR-E* (JRY8994). (C) *HMR-E* DNA recovered from Rap1-Myc chromatin immunoprecipitation. Anti-myc antibody was used to immunoprecipitate DNA cross-linked to Rap1-Myc proteins from strains containing either the native Rap1 binding site at *HMR-E* (JRY9021 and JRY9022 biological replicates) or the genome-wide consensus Rap1 binding site at *HMR-E* (JRY9023 and JRY9024 biological replicates). Cells lacking the myc tag were used as a control.

Given the peculiar conservation of the weak Rap1 binding site at *HMR-E*, we predicted that the genome-wide consensus binding site for Rap1 would not silence the *HMR*-**a1** gene as effectively as the native Rap1 binding site. Hence, we replaced the *HMR-E* variant (AAACCCATAAC) with the genome-wide consensus sequence for the Rap1 protein (ACACCCATACATT). At steady state, the levels of silencing in a strain with the native *HMR-E* were indistinguishable from the *consensus-Rap1-HMR-E* strain (Figure 2A). Because silencing in some contexts is sensitive to carbon sources (Shei and Broach 1995), we also compared the two strains under different carbon sources, and again found no difference (Özaydin 2009).

Recent results on the kinetics of the establishment of silencing indicate that several cell divisions are required to achieve full silencing in cells in which silencing had been previously disrupted. Moreover, some mutations can affect the kinetics but not the level of silencing (Katan-Khaykovich and Struhl 2005; Osborne *et al.* 2009). Steady-state measurements could therefore potentially miss such differences between silencers, particularly because the Rap1 site is important for the initiation of silencing. We tracked the kinetics of establishment of silencing, comparing the native *HMR-E* and the *consensus-Rap1-HMR-E* strains in cells previously treated with nicotinamde, which inhibits silencing by disrupting the catalytic activity of Sir2 (Bitterman *et al.* 2002). The rates at which silencing was established were indistinguishable between the two strains (Figure 2B).

Relative to the strength of silencing at the telomere and at *HML*, silencing at *HMR* is both strong and robust, as many of the mutations that affect silencing at the telomere and at *HML* retain wild-type levels of silencing at *HMR*. Therefore, a subtle difference in Rap1 binding ability might not result in a loss of silencing at that locus. Still, we were curious whether the consensus Rap1 binding site at *HMR-E* was capable of recruiting Rap1 protein to the same level as the wild-type *HMR-E* sequence. To test the level of Rap1 enrichment at *HMR-E* in the two different strains, we used ChIP for DNA associated with Rap1-Myc in those strains. DNA from these enrichments was amplified at the *HMR-E* region, at a positive control region of Telomere VI, and at a negative control region, *SEN1* (Figure 2C). There was no detectable difference between the Rap1 enrichment at *HMR-E* in samples containing the native Rap1 binding site and those with the Rap1 consensus sequence placed at *HMR-E*. These findings indicate that relative to the positive and negative controls, both Rap1 binding sequences were equally capable of localizing Rap1 to the silencer region and of establishing and maintaining functionally silent chromatin.

### The Rap1 consensus binding site at *HMR-E* improved silencing in *sir1*Δ cells

Because both binding sites for Rap1 were capable of mediating silent chromatin formation, those results yielded no insight into what selective pressure may have shaped the *HMR-E* Rap1 binding site sequences. We reasoned that the selective advantage may only be observable in a sensitized context in which small differences in silencer function may be translated into observable differences. To test for small differences in silencing strengths between the two Rap1 binding sites, we performed reverse transcription qPCR in *sir1*Δ mutants containing either version of the silencer. The *sir1*Δ mutation was chosen to optimize the chance for observable phenotypic differences as this mutation does in other contexts (Osborne *et al.* 2009; van Welsem *et al.* 2008). Cells lacking *SIR1* have a partial loss of silencing phenotype at *HMR* with roughly 50% of the transcript level observed in a *sir2Δ* strain (Pillus and Rine 1989; Xu *et al.* 2006). Therefore, slight increases or decreases in expression level could be easily observed.

**Figure 3:**
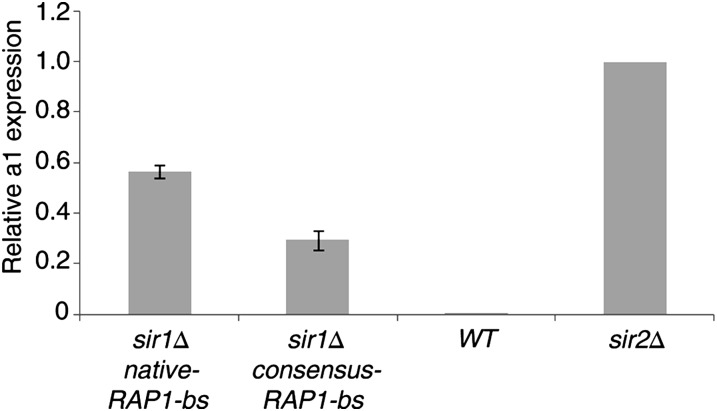
Improved silencing by Rap1 binding site at *HMR-E* in *sir1*Δ cells. Reverse transcription-qPCR measurements of *HMR*-***a****1* transcript levels normalized to *ACT1* control for strains lacking *SIR1* but containing either the native *HMR-E* sequence (JRY9017) or genomic consensus version of the Rap1 binding site (JRY9019). As controls, wild-type cells (JRY3009), and cells lacking *SIR2* (JRY4565) also were tested. Experiments are shown as the average of three triplicate experiments normalized to *sir2*Δ expression levels, with standard error of the mean indicated by error bars.

Again, the consensus Rap1 binding site at *HMR-E* was not defective for silencing. Moreover, the replacement of the native *HMR-E* Rap1 binding site for the consensus sequence actually improved silencing strength, as indicated by a reduced level of ***a****1* expression in the *sir1*Δ background (Figure 3). These results clearly established that the particular variant of the Rap1 binding site at *HMR-E* was not necessary for full silencing function of the *HMR* locus. Thus, collectively the data indicated that the particular Rap1 binding site at silencers did not act as an allosteric effector of silencing and did not evolve for maximal silencing strength.

### No evidence of genome-wide selection against Abf1 and Rap1 binding site co-occurrence

The ability of the genome-wide consensus sequence of the Rap1 binding site to function in *HMR-E*’s role as a silencer underscored the question of how the yeast cell prevents spurious silencing in Rap1 and Abf1-bound regions of the genome. We focused on the Rap1 and Abf1 sites, ignoring the Orc1 binding sites [ARS Consensus Sequence (ACS)] for two reasons. First, the requirement for the ACS is imprecisely specified in the four silencers, with a single exact match or multiple near-matches also present, depending on the silencer. Second, the evolutionary spacing across the *sensu stricto* species between the Rap1 and Abf1 binding sites at *HMR-E* is known, but how close the ACS has to be to either of those sites is unknown (Teytelman *et al.* 2008).

**Figure 4:**
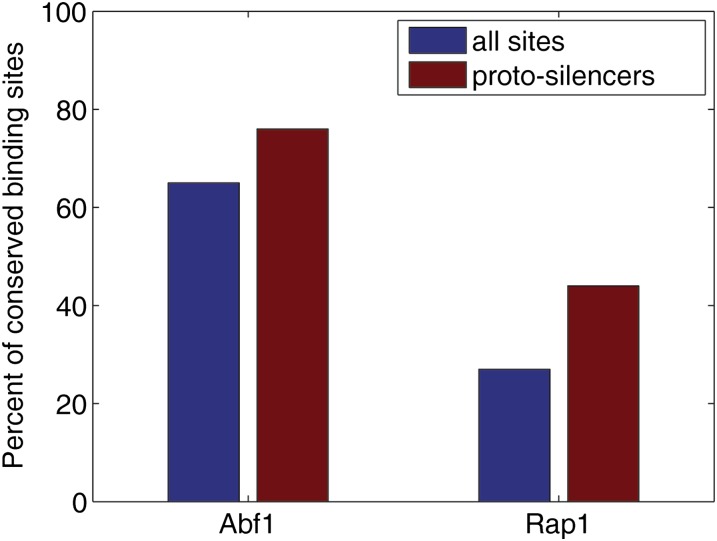
Conservation of Rap1 and Abf1 binding sites across species. Percent of *S. cerevisiae* binding sites conserved in three or more *sensu stricto* species, for Rap1 and Abf1 binding sites. The percent of conserved sites is shown in blue for all genomic matches. Shown in red is the percent for the 25 proto-silencer sites where Abf1 and Rap1 matches are within 50 bp of each other. The differences within Rap1 and Abf1 were not significant by the χ^2^ test at the 0.05 *P*-value cut-off.

On the basis of the *HMR-E* architecture in the *sensu stricto*, 25 potential proto-silencers of *Saccharomyces*, defined as euchromatic intergenic regions in which the Rap1 and Abf1 binding sites occur within 50 base pairs of each other, were identified (Table 3). We then asked whether negative selection against proto-silences could restrict silencing to *HML/HMR*, telomeres, rDNA and subtelomeres. Because the Rap1 genome-wide consensus binding site was fully functional in its silencing role at the *HMR-E*, it was possible that proto-silencers could also nucleate silencing in euchromatin. We reasoned that the binding sites would be less likely to be conserved if their occurrences were deleterious, as would be expected if the potential proto-silencers occasionally silenced adjacent genes. Hence, we measured the conservation of Rap1 and Abf1 binding sites across the *sensu stricto* species, comparing Rap1 and Abf1 binding-site conservation in all intergenic regions to the conservation in the 25 proto-silencers. Conservation was defined as the presence of a binding site in three or more species (MacIsaac *et al.* 2006). The binding sites for both Rap1 and Abf1 in the potential proto-silencers were no less likely to be conserved than genome-wide Rap1 and Abf1 binding sites outside of this context (Figure 4). This result suggested that there was no spurious silencing at the proto-silencers and that existence of such proto-silencers was not deleterious for the cell.

**Table 3:** Loci at which Rap1 and Abf1 binding sites co-occur within 50 base pairs of each other (proto-silencers)

Chromosome	Rap1 binding site	Abf1 binding site
*I*	*141851*	*141829*
*II*	*682048*	*682084*
*IV*	*43804*	*43828*
*IV*	*836178*	*836216*
*V*	*491217*	*491253*
*VI*	*58434*	*58387*
*VII*	*197190*	*197205*
*IX*	*254357*	*254318*
*X*	*651339*	*651294*
*X*	*684313*	*684307*
*XI*	*39028*	*39012*
*XI*	*327627*	*327609*
*XI*	*407935*	*407972*
*XI*	*416723*	*416684*
*XII*	*202816*	*202847*
*XII*	*636493*	*636519*
*XII*	*1064491*	*1064536*
*XIII*	*551528*	*551491*
*XIV*	*57801*	*57820*
*XIV*	*355971*	*355965*
*XV*	*216461*	*216412*
*XV*	*274433*	*274471*
*XV*	*780709*	*780747*
*XV*	*1009784*	*1009821*
*XVI*	*866585*	*866602*

*S. cerevisiae* regions in which Rap1 and Abf1 binding sites are within 50 base pairs of each other. The locations of matches to the Rap1 and Abf1 binding profile are from the MacIsaac *et al.*, 2006 study (Macisaac *et al.* 2006).

**Figure 5:**
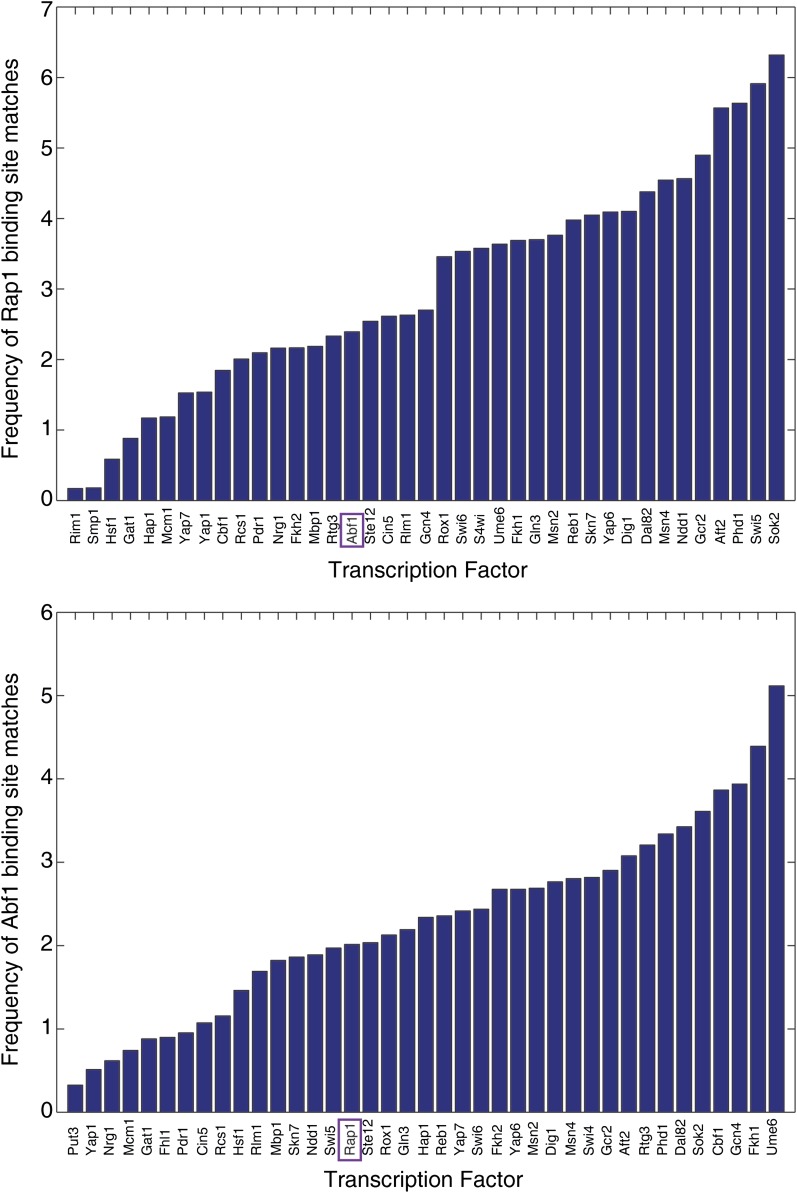
Binding site frequency in transcription factor-bound intergenic regions. (Top) The frequency of Rap1 binding site occurrence within the intergenic regions bound by the indicated transcription factors; calculated as the number of Rap1 sites, divided by the sum of the lengths of all intergenic regions containing the transcription factor (see *Materials and Methods* for details). The purple rectangle highlights the Rap1 binding site frequency in Abf1-bound regions. (Bottom) Same as in the top panel but for Ab11 binding sites.

As an additional test of selection against co-occurrence of Rap1 and Abf1 binding sites, we tested for signs of such negative selection by asking whether Abf1 binding sites occur less frequently in intergenic regions with known Rap1 binding, and vice versa, compared to regions bound by other transcription factors. In line with our previous results, the frequency of Abf1 binding sites was not decreased in Rap1-bound intergenic regions, nor was the frequency of Rap1 binding sites in Abf1-bound regions (Figure 5).

## Discussion

For budding yeasts, silencing is a tricky balancing act. On one hand, the transcription of the *HML* and *HMR* loci must be robustly repressed at all times. On the other hand, silenced chromatin must be prevented from ectopic formation in most of the genome. This conflict is particularly challenging because the silencers use DNA-binding proteins that are important in euchromatin function at other regions of the genome. The problem is conceptually similar to the need to have one and only one centromere per chromosome, avoiding neocentromere activation at other sites [reviewed in (Sullivan *et al.* 2001)].

Strikingly, a Rap1 binding site in *HMR-E*, although a poor match to the Rap1 binding profile, was conserved in five species despite being located in the midst of a rapidly evolving region. This apparent paradox suggested the possibility that the role of Rap1 could be tailored to silencing or transcription activation by the particular sequence of its binding site within a silencer. However, the data presented here established clearly that a consensus version of the Rap1 site at the *HMR-E* silencer could stably maintain silencing in a population of cells, could establish silencing as quickly as a natural silencer, and was at least as robust to sensitizing mutations as a natural silencer.

These results were puzzling, considering the ability of Abf1 and Rap1 bindings sites to establish silencing at *HMR-E* in the absence of an ORC binding site, and the ability of multiple Rap1 sites to nucleate telomeric silencing (Brand *et al.* 1987; Cockell *et al.* 1995; Hecht *et al.* 1996). If the consensus binding sites for Rap1 and Abf1 can initiate silencing, how does the cell prevent ectopic silencing in the many intergenic regions in which Rap1 and Abf1 sites co-occur? We investigated this question by analyzing whether there is purifying selection against co-occurrence of Rap1 and Abf1 motifs near each other. Our results showed no evidence of deleterious ectopic Sir-protein recruitment, as measured by the absence of a signal of selection against adjacent Rap1-Abf1 binding sites.

Our work highlights a missing dimension to an understanding of the selective forces acting on the anatomy of silencers. We conclude that the selective force for the retention of the particular Rap1 site in the *HMR-E* silencer is apparently unrelated to silencing, with some other function providing the selective pressure. Silencers associate with cohesins (Chang *et al.* 2005), but many other sites do as well, making that explanation of selective force unlikely. Silencer function is required in mating-type interconversion to distinguish donor cassettes from recipient loci through the protection of the HO cut site at *HML* and *HMR*, which would seem to have the same requirements as silencing *per se*. However, the pattern of mating-type interconversion is highly regulated, and only partially explained by the recombinational enhancer near *HML*. It is conceivable that some aspect of the way that Rap1 binds a silencer plays a nuanced but sufficiently compelling contribution to interconversion to explain the enigma of the Rap1 binding site conservation (Haber 2012).
